# Higher proportion of non-classical and intermediate monocytes in newly diagnosed multiple myeloma patients in Egypt: A possible prognostic marker

**DOI:** 10.4102/ajlm.v10i1.1296

**Published:** 2021-08-25

**Authors:** Asmaa M. Zahran, Hanaa Nafady-Hego, Sawsan M. Moeen, Hanan A. Eltyb, Mohammed M. Wahman, Asmaa Nafady

**Affiliations:** 1 Department of Clinical Pathology, South Egypt Cancer Institute, Assiut University, Assiut, Egypt; 2 Department of Microbiology and Immunology, Faculty of Medicine, Assiut University, Assiut, Egypt; 3 Department of Internal Medicine, Clinical Haematology Unit, Faculty of Medicine, Assiut University, Assiut, Egypt; 4 Department of Medical Oncology, South Egypt Cancer Institute, Assiut University Assiut, Egypt; 5 Department of Clinical Oncology, South Valley University, Qena, Egypt; 6 Department of Clinical and Chemical Pathology, Qena Faculty of Medicine, South Valley University, Qena, Egypt

**Keywords:** multiple myeloma, classical monocytes, intermediate monocytes, non-classical monocytes, flow cytometry

## Abstract

**Background:**

Interaction between multiple myeloma (MM) cells and proximal monocytes is expected during plasma cell proliferation. However, the role of monocyte subsets in the disease progression is unknown.

**Objective:**

This study evaluated circulating monocyte populations in MM patients and their correlation with disease severity.

**Methods:**

Peripheral monocytes from 20 patients with MM attending Assiut University Hospital in Assiut, Egypt, between October 2018 and August 2019 were processed using a flow cytometry procedure and stratified using the intensity of expression of CD14 and CD16 into classical (CD16^−^CD14^++^), intermediate (CD16^+^CD14^++^), and non-classical (CD16^++^CD14^+^) subsets. The data were compared with data from 20 healthy control participants with comparable age and sex.

**Results:**

In patients with MM, the percentage of classical monocytes was significantly lower (mean ± standard error: 77.24 ± 0.66 vs 83.75 ± 0.5), while those of non-classical (12.44 ± 0.5 vs 8.9 ± 0.34) and intermediate (10.3 ± 0.24 vs 7.4 ± 0.29) monocytes were significantly higher when compared with those of controls (all *p* < 0.0001). Proportions of non-classical and intermediate monocytes correlated positively with serum levels of plasma cells, M-protein, calcium, creatinine and lactate dehydrogenase, and correlated negatively with the serum albumin level. Proportions of classical monocytes correlated positively with albumin level and negatively correlated with serum levels of M-protein, plasma cells, calcium, creatinine, and lactate dehydrogenase.

**Conclusion:**

Circulating monocyte subpopulations are skewed towards non-classical and intermediate monocytes in MM patients, and the intensity of this skewness increases with disease severity.

## Introduction

Multiple myeloma (MM) is a cancer that affects B-cells, particularly plasma cells, leading to their expansion and the excessive production of immunoglobulins. Malignant plasma cells may control the quality of the immune cells in the bone marrow and produce immunoglobulins that can act as tumour-associated antigens which are further processed by antigen-presenting cells to stimulate specific anti-tumour T-cell responses.^1^ Monocytes and macrophages play a fundamental role in this inflammatory response, possibly through bone marrow expansion and cytokine production in myeloma patients.^2,3,4,5^ This is corroborated by findings from in-vitro studies.^6,7^ The increased peripheral monocytes in MM patients may inhibit antitumor immune responses and increase tumour aggression.^2,3^ Human peripheral blood monocytes are a heterogeneous population, as they differ in their phenotype, surface marker expression, and function.^8^ They can be classified based on the fluorescent intensities of CD16 and CD14 into three distinct populations, including classical monocytes (CD16^−^CD14^++^), intermediate monocytes (CD16^+^CD14^++^), and non-classical monocytes (CD16^++^CD14^+^).^9^ Circulating non-classical monocytes have been observed to increase in some autoimmune diseases such as lupus and rheumatoid arthritis,^10,11^ and the frequency of intermediate monocytes has been associated with bone erosion in patients with psoriatic arthritis.^12^ In oncology settings, the intermediate monocytes have been reported to play a crucial role in patients with MM,^9,13^ and the proportion of intermediate monocytes increased proportionately with the tumour load.^14,15^ Myeloma cells produce toll-like receptor ligands that cause the expansion of non-classical monocytes in MM patients.^16^ In addition, circulating monocytes correlate with the well-known prognostic factors for MM, and evaluating monocyte subpopulations can enhance our understanding of MM mechanisms and aid patient follow-up. Although bone marrow aspirates and trephine biopsies provide high diagnostic yields for MM detection,^17^ finding a less invasive diagnostic procedure such as flow cytometry will be a clinical breakthrough.

This study aimed to describe the immunophenotypic profile of the peripheral blood monocytes in newly diagnosed MM patients at the Assiut University Hospital, Assiut, Egypt.

## Methods

### Ethical considerations

The Human Research Ethics Committee of the South Egypt Cancer Institute, Assiut University, approved this study (SECI-IRB-IORG0006563, 37:10/1/2018). Written informed consent was obtained from patients and healthy volunteers in accordance with the Declaration of Helsinki. Patients and controls provided written informed consent and participated in the study after study clarification. Patients got the maximum care, and the study procedures were in accordance with relevant guidelines and the Declaration of Helsinki. Investigators and researchers collected data using paper data collection forms. Patients were identified by serial study codes, which were linked with patient names and hospital file numbers on a separate sheet and stored in a locked safe cabinet. Data analysis was performed on the de-identified data.

### Study population

A prospective case-control study was conducted at the Clinical Haematology Unit, Internal Medicine Department, Assiut University Hospital, and Medical Oncology Department, South Egypt Cancer Institute, Assiut University, in Assiut, Egypt, from October 2018 to August 2019. Twenty newly diagnosed untreated MM patients diagnosed according to the World Health Organization criteria for MM, and 20 matched (age and sex) healthy volunteers were included in this study. Patients who were under treatment or refused to participate were not included.

Multiple myeloma patients were classified using the Durie-Salmon staging system^18^ that assesses tumour burden based on three laboratory parameters and evaluates bone involvement based on a radiographic skeletal survey. Patients who had haemoglobin levels over 10 g/dL, normal serum calcium or less than 10.5 mg/dL, normal bone X-ray or solitary lytic lesion, monoclonal immunoglobulin G under 5 g/dL, immunoglobulin A under 3 g/dL, and Bence Jones protein under 4 g/24 h were classified as stage I. Stage II patients were patients with a moderate number of myeloma cells that did not meet either stage I or stage III criteria. Stage III patients were those who met one or more of the following criteria: haemoglobin under 8.5 g/dL, serum calcium value over 12 mg/dL, advanced lytic bone lesions, monoclonal immunoglobulin G over 7 g/dL, immunoglobulin A over 5 g/dL, and Bence Jones protein over 12 g/24 h.

As the Durie-Salmon scoring may not provide good prognostic discrimination, the International Staging System was used in this study to group MM patients according to the serum β_2_-microglobulin (β_2_M) and serum albumin into 3 stages: stage I characterised by serum β_2_M less than 3.5 mg/L and serum albumin 3.5 g/dL or higher, stage II in which serum β_2_M was less than 3.5 mg/L and serum albumin was less than 3.5 g/dL or β_2_M was between 3.5 mg/L and 5.5 mg/L, and stage III in which serum β_2_M was 5.5 mg/L or higher.^19^

Patient data were also collected for the study; these included age, sex, and clinical history of bone pain, pathological fracture, weakness and fatigue, recurrent infection, manifestation of hypercalcemia such as thirst, polyuria, manifestation of hyperviscosity (blurring of vision, mental confusion), bleeding tendency, and neuropathy. Clinical signs of anaemia, oedema, and renal impairment were carefully assessed in all patients. A radiographic skeletal survey of the skull and long bones was also done to detect lytic bone lesions, pathological fractures, and abdominal ultrasound.

Complete blood count with differential was done using the Cell-Dyn Ruby fully automated blood counter (CELL-DYN Ruby System, Abbott Diagnostics, Chicago, Illinois, United States; serial number: 36026BG). Examination of peripheral blood smear for rouleaux formation was done as part of routine care. Blood urea, serum creatinine, total protein, albumin, and lactate dehydrogenase (LDH) tests were done using the Cobas Integra 400 automated chemical analyser (Roche Diagnostics GmbH, Mannheim, Germany; serial number: 500558). Serum protein electrophoresis with immunofixation was performed to identify monoclonal protein (M-protein) types among patients at diagnosis using the *Pretty Interlab* device (Interlab, Rome, Italy; serial number: 38405301). Bone marrow aspiration and biopsy to evaluate and quantify bone marrow plasma cell infiltration were done on all recruited patients. All these investigations were done as routine services for newly diagnosed MM cases.

### Flow cytometric detection of monocytes subtypes

**FIGURE 1:**
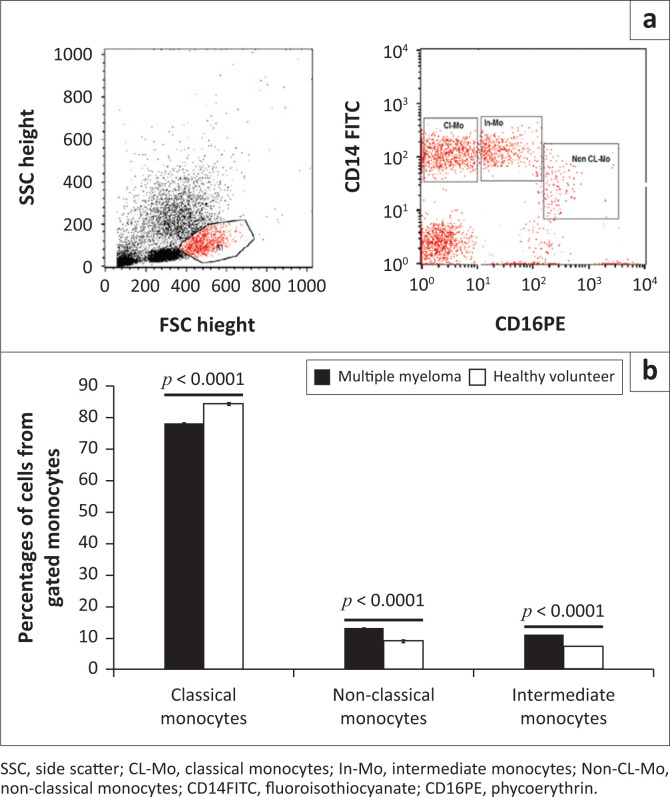
Monocyte subsets in multiple myeloma patients and healthy controls at Assiut University, Assiut, Egypt from October 2018 to August 2019. (a) Flow cytometric detection of monocytes subsets. Forward scatter and side scatter dot was used to detect monocyte region (R1) (left). CD14-fluoroisothiocyanate- and CD16-phycoerythrin-positive cells were identified among monocytes and classified as CL-Mo (classical monocytes, CD14^++^CD16^-^), In-Mo (intermediate monocytes (CD14^++^CD16^+^) and Non-CL-Mo (non-classical monocytes CD14^+^CD16^++^) (right). (b) The frequency of the monocytes subsets varies significantly between multiple myeloma patients and healthy controls (classical monocytes: mean ± standard error 77.24 ± 0.66 in multiple myeloma patients vs 83.75 ± 0.5 among controls, *p* < 0.0001; non-classical monocytes: 12.44 ± 0.5 in multiple myeloma patients vs 8.9 ± 0.34 among controls, *p* < 0.0001; and intermediate monocytes: 10.3 ± 0.24 in multiple myeloma patients vs 7.4 ± 0.29 among controls, *p* < 0.0001).

Fifty microlitres of remnant routine complete blood count samples were added to 5 µL of CD14 fluoroisothiocyanate-conjugated and CD16 phycoerythrinconjugated (Becton Dickinson [BD] Biosciences, San Jose, California, United States). After 15 min of incubation at room temperature in the dark, lysis and washing of erythrocytes was done. The cells were then re-suspended in phosphate-buffered saline, and cells were acquired using the BD FACSCalibur flow cytometry system (BD Biosciences, San Jose, California, United States), with data analysis done using the CellQuest software (BD Biosciences, San Jose, California, United States). An anti-human immunoglobulin G isotype-matched negative control was used for each flow cytometry experiment. Forward and side scatter histograms were used to define monocytes. Afterwards, the expression of CD14 and CD16 were assessed in the monocyte population. The classical subset was CD14^++^CD16^-^, intermediate subset was CD14^++^CD16^+^, and non-classical subset was CD14^+^CD16^++^ (Figure 1). Monocyte subset data are expressed as percentages of the total monocytes.

### Statistical analysis

We used Statistical Package for the Social Sciences version 21 (IBM Corporation, Armonk, New York, United States) for data analysis. Quantitative data are presented as mean ± standard error while qualitative data are presented as frequencies and percentages. Mann-Whitney test analysis was performed to determine the statistical significance and differences between the study groups. Spearman’s correlation was performed to determine the association between monocytes subtypes and laboratory parameters. Results were considered significant if *p* was 0.05 or less.

## Results

### Baseline characteristics of newly diagnosed multiple myeloma patients and healthy controls

The mean age of the MM patients was 6.35 ± 4.07 years, and the number of men (13) was double that of women (7) (Table 1). The proportion of plasma cells in bone marrow was 39.7 ± 3.7% and that of the monoclonal band (M-protein) was 4.44 ± 2.8 g/dL.

**TABLE 1:** Baseline characteristics of studied newly diagnosed multiple myeloma patients and healthy controls, Assiut, Egypt, October 2018 – August 2019.

Variable	Multiple myeloma patients (*n* = 20)			Healthy controls (*n* = 20)			*p*
	Mean ± standard error	*n*	%	Mean ± standard error	*n*	%	
Age (years)	63.5 ± 4.07	-	-	61 ± 3.5	-	-	-
Age range (years)	38–71	-	-	36–69	-	-	-
**Sex**							
Men	-	13	-	-	12	-	-
Women	-	7	-	-	8	-	-
Total leucocyte count (10^9^/L)	8.4 ± 1.9	-	-	7.9 ± 1.9	-	-	0.281
Platelets (10^9^/L)	199 ± 41	-	-	227 ± 45	-	-	0.163
Haemoglobin (g/dL)	9.3 ± 2.4	-	-	12.8 ± 1.97	-	-	0.045
Total protein (g/dL)	7.15 ± 0.8	-	-	7.08 ± 0.7	-	-	0.496
Albumin (g/dL)	2.46 ± 0.9	-	-	4.63 ± 1.1	-	-	< 0.0001
Calcium (mg/dL)	11.75 ± 0.4	-	-	8.8 ± 0.1	-	-	< 0.0001
Urea (mg/dL)	43.98 ± 3.25	-	-	39.3 ± 1.5	-	-	0.194
Creatinine (mg/dL)	1.9 ± 0.2	-	-	0.97 ± 0.1	-	-	< 0.0001
Lactate dehydrogenase (U/L)	886.86 ± 92.6	-	-	155.80 ± 8.23	-	-	< 0.0001
M-protein (g/dL)	4.44 ± 2.8	-	-	-	-	-	-
Bone marrow plasma cells (%)	39.7 ± 3.7	-	-	-	-	-	-
Classical monocytes (%)	77.24 ± 0.66	-	-	83.75 ± 0.5	-	-	< 0.0001
Non-classical monocytes (%)	12.44 ± 0.5	-	-	8.9 ± 0.34	-	-	< 0.0001
Intermediate monocytes (%)	10.3 ± 0.24	-	-	7.4 ± 0.29	-	-	< 0.0001
**International Staging System staging**							
Stage I	-	14	70	-	-	-	-
Stage II	-	4	20	-	-	-	-
Stage III	-	2	10	-	-	-	-
**Durie-Salmon staging**							
Stage I	-	12	60	-	-	-	-
Stage II	-	6	30	-	-	-	-
Stage III	-	2	10	-	-	-	-

U/L, units per litre.

Laboratory investigations showed that platelet counts, total leukocytic counts, serum total protein, and urea levels were comparable between patients and healthy controls (*p* > 0.05).

Multiple myeloma patients had significantly reduced haemoglobin (9.3 ± 2.4 g/dL in MM vs 12.8 ± 1.97 g/dL in healthy controls; *p* = 0.045) and serum albumin (2.46 ± 0.9 g/dL in MM patients vs 4.63 ± 1.1 g/dL in healthy controls; *p* < 0.0001) when compared to healthy controls.

On the other hand, after comparing the mean levels of serum calcium, LDH, and serum creatinine between MM patients and healthy controls, we found statistically significant increased levels in MM patients (calcium: 11.75 ± 0.4 mg/dL in MM patients vs 8.8 ± 0.1 mg/dL in healthy controls, *p* < 0.0001; LDH: 886.86 ± 92.6 units per litre in MM vs 155.8 ± 8.23 units per litre in controls, *p* < 0.0001; serum creatinine: 1.9 ± 0.2 mg/dL in MM patients vs 0.97 ± 0.1 mg/dL in healthy controls, *p* < 0.0001).

Based on the Durie-Salmon staging system, 12 (60%) of the 20 newly diagnosed untreated MM patients had stage I disease, 6/20 patients (30%) had stage II disease, and 2/20 patients (10%) had stage III disease. Whereas according to the International Staging System, 70% (*n* = 14) of MM patients had stage I disease, 20% (*n* = 4) had stage II, and 10% (*n* = 2) had stage III.

### Phenotypic characterisation of monocytes in multiple myeloma patients

Monocytes present in the peripheral blood displayed a range of intensities of CD16 and CD14 expression. They were divided into classical (CD16^−^CD14^++^), intermediate (CD16^+^CD14^++^), and non-classical (CD16^++^CD14^+^) based on these ranges.

In healthy controls, classical monocytes represented the major component among total monocytes (83.8%), followed by non-classical monocytes (8.9%) and intermediate monocytes (7.4%).

Multiple myeloma patients had statistically significant lower proportions of classical monocytes (77.24% ± 0.66 in MM patients vs 83.75% ± 0.5 in controls), and higher proportions of non-classical (12.44% ± 0.5 in MM patients vs 8.9% ± 0.34 in controls) and intermediate (10.3% ± 0.24 in MM patients vs 7.4% ± 0.29 in controls) monocytes compared to healthy controls (all with *p* < 0.0001) (Figure 1).

### Correlation between monocyte subsets and laboratory parameters in newly diagnosed untreated multiple myeloma patients

**FIGURE 2:**
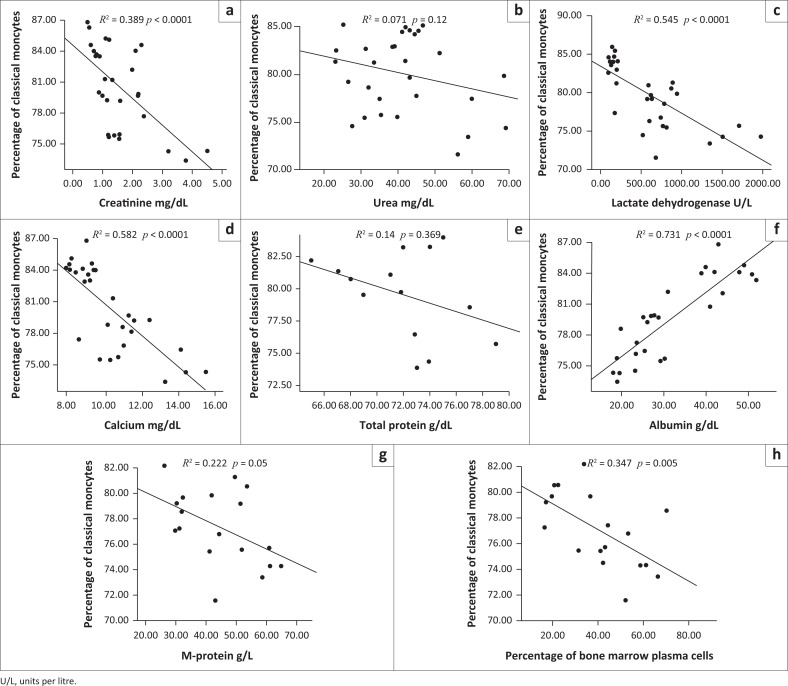
Correlation of classical monocytes with laboratory parameters in multiple myeloma patients and healthy controls at Assiut University, Assiut, Egypt, from October 2018 to August 2019. The percentage of classical monocytes (CD16^−^CD14^++^) and (a) creatinine, (b) urea, (c) lactate dehydrogenase, (d) calcium, (e) total protein, (f) albumin, (g) M-protein, and (h) bone marrow plasma cells % were correlated using Spearman’s correlation test and correlated positively with level of serum albumin (*R*^2^ = 0.731, *p* < 0.0001) (f), and correlated negatively with levels of serum creatinine (*R*^2^ = 0.389, *p* < 0.0001) (a), lactate dehydrogenase (*R*^2^ = 0.545, *p* < 0.0001) (e), calcium (*R*^2^ = 0.582, *p* < 0.0001) (d), M-protein (*R*^2^ = 0.222, *p* = 0.05) (g), and the percentage of bone marrow plasma cells (*R*^2^ = 0.347, *p* = 0.005) (h).

In newly diagnosed untreated MM patients, the proportion (%) of classical monocytes correlated positively with the serum albumin level (*R*^2^ = 0.731; *p* < 0.0001), and correlated negatively with serum levels of serum creatinine (*R*^2^ = 0.389, *p* < 0.0001), LDH (*R*^2^ = 0.545, *p* < 0.0010), calcium (*R*^2^ = 0.582, *p* < 0.0001), M-protein (*R*^2^ = 0.222, *p* = 0.005), and with the percentage of bone marrow plasma cells (*R*^2^ = 0.347, *p* < 0.0001), while urea and total protein did not show significant correlation (Figure 2).

**FIGURE 3:**
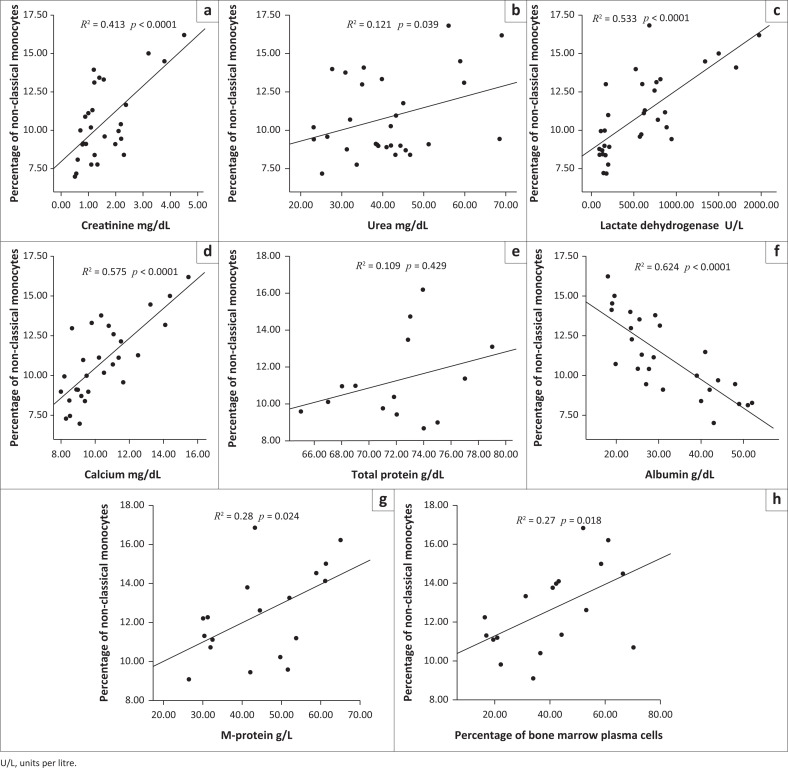
Correlation of non-classical monocytes with laboratory parameters in multiple myeloma patients and healthy controls at Assiut University, Assiut, Egypt, from October 2018 to August 2019. The percentage of non-classical monocytes (CD16^++^CD14^+^) and (a) creatinine, (b) urea, (c) lactate dehydrogenase, (d) calcium, (e) total protein, (f) albumin, (g) M-protein, and (h) bone marrow plasma cells % were correlated using Spearman’s correlation test and correlated negatively with level of serum albumin (*R*^2^ = 0.624, *p* < 0.0001) (f), and correlated positively with higher levels of creatinine (*R*^2^ = 0.413, *p* < 0.0001) (a), lactate dehydrogenase (*R*^2^ = 0.533, *p* < 0.0001) (c), serum calcium (*R*^2^ = 0.575, *p* < 0.0001) (d), M-protein (*R*^2^ = 0.28, *p* = 0.024) (g), and the percentage of bone marrow plasma cells (*R*^2^ = 0.27, *p* = 0.018) (h).

In contrast, the proportion (%) of non-classical monocytes in newly diagnosed untreated MM patients correlated negatively with the serum albumin level (*R*^2^ = 0.624, *p* < 0.0001), and correlated positively with serum levels of creatinine (*R*^2^ = 0.413, *p* < 0.0001), LDH (*R*^2^ = 0.533, *p* < 0.0001), calcium (*R*^2^ = 0.575, *p* < 0.0001), M-protein (*R*^2^ = 0.28, *p* = 0.024), and with the percentage of bone marrow plasma cells (*R*^2^ = 0.27, *p* = 0.018), while urea and total protein did not show significant correlation (Figure 3).

**FIGURE 4:**
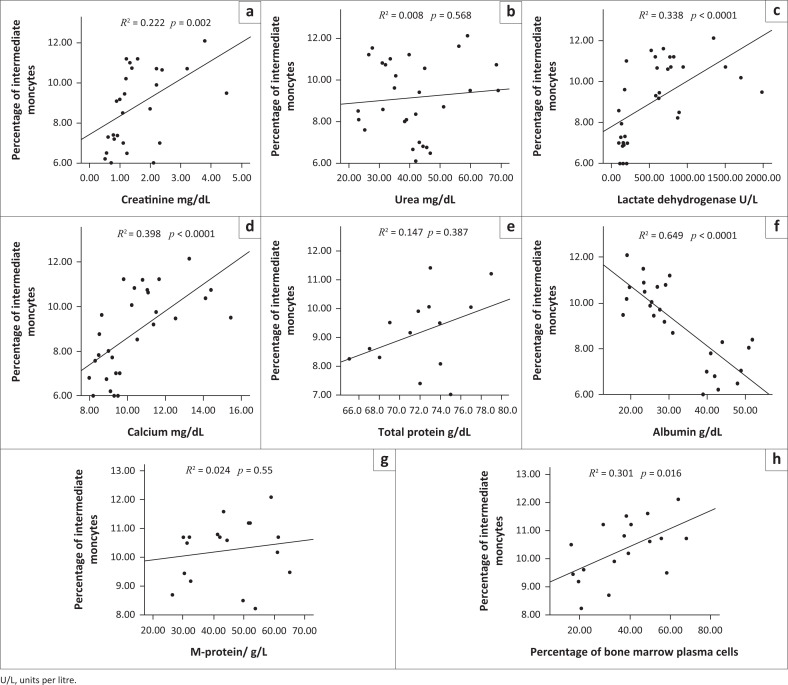
Correlation of intermediate monocytes with laboratory parameters in multiple myeloma patients and healthy controls at Assiut University, Assiut, Egypt, from October 2018 to August 2019. The percentage of intermediate monocytes (CD16^+^CD14^++^) and (a) creatinine, (b) urea, (c) lactate dehydrogenase, (d) calcium, (e) total protein, (f) albumin, (g) M-protein, and (h) bone marrow plasma cells % were correlated using Spearman’s correlation test and correlated negatively with level of serum albumin (*R*^2^ = 0.649, *p* < 0.0001) (f), and correlated positively with levels of serum creatinine (*R*^2^ = 0.222, *p* = 0.002) (a), lactate dehydrogenase (*R*^2^ = 0.338, *p* < 0.0001) (c), calcium (*R*^2^ = 0.398, *p* < 0.0001) (d), and the percentage of bone marrow plasma cells (*R*^2^ = 0.301, *p* = 0.016) (h).

Similarly, the proportion (%) of intermediate monocytes in newly diagnosed untreated MM patients correlated negatively with serum albumin level (*R*^2^ = 0.649, *p* < 0.0001) and correlated positively with the serum levels of creatinine (*R*^2^ = 0.222, *p* = 0.002), LDH (*R*^2^ = 0.338, *p* < 0.0001), calcium (*R*^2^ = 0.398, *p* < 0.0001), and with the percentage of bone marrow plasma cells (*R*^2^ = 0.301, *p* = 0.016), while urea, total protein, and M-protein did not show significant correlation (Figure 4).

## Discussion

This study found that the percentage of classical monocytes was significantly lower in patients with MM compared to controls, while the percentages of non-classical and intermediate monocytes were significantly higher. Moreover, the proportions of non-classical and intermediate monocytes were positively correlated with serum levels of plasma cells, M-protein, calcium, creatinine, and lactate dehydrogenase, and correlated negatively with the serum albumin level. Proportions of classical monocytes correlated positively with albumin level and correlated negatively with serum levels of M-protein, plasma cells, calcium, creatinine, and lactate dehydrogenase.

Multiple myeloma is a plasma cell cancer that exploits monocytes and macrophages for growth and expansion^16^ through the production of inflammatory interleukins such as IL-6 that promote persistence of malignant plasma cells^4,16^ and enhance tumour progression.^5^ Monocytes,^20^ especially non-classical monocytes,^16^ increase in MM patients.

In this study, we analysed circulating monocytes in 20 newly identified untreated MM patients to discover their role in disease progression and postulate their possible use as a prognostic factor during patient follow-up. We found significantly increased proportions of non-classical (CD16^++^ CD14^+^) and intermediate (CD16^+^ CD14^++^) circulating monocytes in newly diagnosed untreated MM patients compared to healthy controls. Our results are similar to those of a study in 2015 in France that found an increased frequency of CD14^+^/^++^ CD16^+^ monocytes in 20 MM patients compared to healthy volunteers; however, they did not find a correlation between the monocyte subsets and circulating or bone marrow plasma cell counts.^15^ Similarly, a study in Norway in 2015 found that the proportion of non-classical monocytes increased with tumour load in MM patients (*n* = 12) and suggested that non-classical monocytes could maintain the development of myeloma cells.^16^ However, the study noted that the increased proportion of non-classical monocytes might be caused by numerous mechanisms. By the end of the study, the investigators concluded that the non-classical subset of monocytes in patients with MM are functional monocytes that could be stimulated to produce cytokines, and may enhance myeloma expansion, contributing to the disease process.

Furthermore, malignant plasma cells yield increased levels of transforming growth factor beta 1,^21^ which hypothetically increases CD16 expression. Therefore, it is probable that in patients with high plasma cell levels, CD14-high cells under the effect of transforming growth factor beta 1 settle into the CD16^+^CD14^dim^ cell regions.

The different monocyte subset proportions between MM patients and healthy controls could be explained by several mechanisms. Nucleic acids and immune complexes produced during disease mainly encourage the production of intermediate and non-classical monocyte subsets, and these yield interleukins to maintain myeloma growth in MM patients.^14^ Also, higher proportions of the non-classical subset of monocytes could be produced by numerous mechanisms such as stimulation by M-protein, which is produced by the malignant plasma cells,^16^ as myeloma cells secrete higher quantities of immunoglobulins.^9^ Further studies are necessary to understand the mechanisms behind these skewed monocyte subpopulation frequencies and whether it is a cause of disease severity.

Multiple myeloma patients had lower levels of haemoglobin, albumin, and calcium when compared to healthy control volunteers (*p* = 0.045, *p* < 0.0001, and *p* < 0.0001). Parts of these findings are in agreement with a study conducted in Austria in 2010 which reported that most MM cases had anaemia of variable severity.^22^

Similarly, in a study conducted in the United States in 1982,^23^ we found no differences between the platelet count and total leukocytic count in MM cases and healthy controls. The urea levels were comparable among MM patients and controls, while the serum creatinine levels were significantly higher in MM patients compared to controls (*p* < 0.0001); this is in contrast to previous reports that found variable degrees of renal impairment in patients with MM.^24,25^ Lactate dehydrogenase was significantly increased in MM patients versus controls (*p* < 0.0001), as previously reported.^26^

The proportion of plasma cells in bone marrow was 39.7 ± 3.7% and that of the monoclonal band (M-protein) was 44.4 ± 2.8 g/L, comparable with that of previous reports.^16^

### Limitations

One limitation of this study was the small sample size, but these initial results provide new perspectives on the diagnosis and monitoring of MM.

### Conclusion

The higher frequencies of non-classical and intermediate monocytes in newly diagnosed untreated MM patients suggest a possible role of these cells rather than classical monocytes in the disease process. The participation of these monocyte subsets in MM pathogenesis should thus be part of future investigations as they may provide important information for the diagnosis and follow-up of MM patients. More studies are needed to address the possibility of using flow cytometry results as a primary staging criterion.
