# Corrigendum: Higher proportion of non-classical and intermediate monocytes in newly diagnosed multiple myeloma patients in Egypt: A possible prognostic marker

**DOI:** 10.4102/ajlm.v11i1.1713

**Published:** 2022-03-25

**Authors:** Asmaa M. Zahran, Hanaa Nafady-Hego, Sawsan M. Moeen, Hanan A. Eltyb, Mohammed M. Wahman, Asmaa Nafady

**Affiliations:** 1 Department of Clinical Pathology, South Egypt Cancer Institute, Assiut University, Assiut, Egypt; 2 Department of Microbiology and Immunology, Faculty of Medicine, Assiut University, Assiut, Egypt; 3 Department of Internal Medicine, Clinical Haematology Unit, Faculty of Medicine, Assiut University, Assiut, Egypt; 4 Department of Medical Oncology, South Egypt Cancer Institute, Assiut University, Assiut, Egypt; 5 Department of Clinical Oncology, South Valley University, Qena, Egypt; 6 Department of Clinical and Chemical Pathology, Qena Faculty of Medicine, South Valley University, Qena, Egypt

In the published article, Zahran AM, Nafady-Hego H, Moeen SM, Eltyb HA, Wahman MM, Nafady A. Higher proportion of non-classical and intermediate monocytes in newly diagnosed multiple myeloma patients in Egypt: A possible prognostic marker. Afr J Lab Med. 2021;10(1), a1296. https://doi.org/10.4102/ajlm.v10i1.1296, there was an error on page 3 under the ‘Results’ section. The mean age of the MM patients should be 63.5 as per Table 1 instead of 6.35. The first paragraph under the ‘Results’ section is updated to:

## Results

### Baseline characteristics of newly diagnosed multiple myeloma patients and healthy controls

The mean age of the MM patients was 63.5 ± 4.07 years, and the number of men (13) was double that of women (7) (Table 1). The proportion of plasma cells in bone marrow was 39.7% ± 3.7% and that of the monoclonal band (M-protein) was 4.44 g/dL ± 2.8 g/dL.

The authors apologise for this error. The correction does not change the study’s findings of significance or overall interpretation of the study’s results or the scientific conclusions of the article in any way.

